# Stably engineered nanobubbles and ultrasound - An effective platform for enhanced macromolecular delivery to representative cells of the retina

**DOI:** 10.1371/journal.pone.0178305

**Published:** 2017-05-25

**Authors:** Sachin S. Thakur, Micheal S. Ward, Amirali Popat, Nicole B. Flemming, Marie-Odile Parat, Nigel L. Barnett, Harendra S. Parekh

**Affiliations:** 1 School of Pharmacy, The University of Queensland, Woolloongabba, Queensland, Australia; 2 Mater Research Institute, Translational Research Institute, The University of Queensland, Woolloongabba, Queensland, Australia; 3 School of Medicine, The University of Queensland, Herston, Queensland, Australia; 4 Queensland Eye Institute, South Brisbane, Queensland, Australia; 5 UQ Centre for Clinical Research, The University of Queensland, Herston, Queensland, Australia; 6 School of Biomedical Sciences, Queensland University of Technology, Brisbane Queensland, Australia; University of Florida, UNITED STATES

## Abstract

Herein we showcase the potential of ultrasound-responsive nanobubbles in enhancing macromolecular permeation through layers of the retina, ultimately leading to significant and direct intracellular delivery; this being effectively demonstrated across three relevant and distinct retinal cell lines. Stably engineered nanobubbles of a highly homogenous and echogenic nature were fully characterised using dynamic light scattering, B-scan ultrasound and transmission electron microscopy (TEM). The nanobubbles appeared as spherical liposome-like structures under TEM, accompanied by an opaque luminal core and darkened corona around their periphery, with both features indicative of efficient gas entrapment and adsorption, respectively. A nanobubble +/- ultrasound sweeping study was conducted next, which determined the maximum tolerated dose for each cell line. Detection of underlying cellular stress was verified using the biomarker heat shock protein 70, measured before and after treatment with optimised ultrasound. Next, with safety to nanobubbles and optimised ultrasound demonstrated, each human or mouse-derived cell population was incubated with biotinylated rabbit-IgG in the presence and absence of ultrasound +/- nanobubbles. Intracellular delivery of antibody in each cell type was then quantified using Cy3-streptavidin. Nanobubbles and optimised ultrasound were found to be negligibly toxic across all cell lines tested. Macromolecular internalisation was achieved to significant, yet varying degrees in all three cell lines. The results of this study pave the way towards better understanding mechanisms underlying cellular responsiveness to ultrasound-triggered drug delivery in future *ex vivo* and *in vivo* models of the posterior eye.

## Introduction

Pathologies of the retina continue to pose an ominous burden on healthcare systems globally with conditions such as age-related macular degeneration (AMD), glaucoma and diabetic retinopathies (DR) listed among the top 10 priority eye diseases by the World Health Organization [1]. While various promising therapeutic agents have been developed in recent years, an overwhelming bottleneck to their utility remains an inability to preferentially deliver them into target tissue/cells of the posterior eye with any level of precision or accuracy [2]. This is in part due to the remote and highly inaccessible location of the affected retinal tissue, which is multi-layered and comprising many associated protective barriers.

To address this ultrasound-assisted drug delivery has emerged as a safe and practical approach by which molecular permeation can be enhanced both into and beyond cells and tissues of interest [3–6]. While it is well-reported that sonoporation/sonophoresis alone yields modest increases in molecular permeation, combining this with ultrasound responsive vectors, such as micro/nanobubbles, leads to significant improvements in the rate and extent of payload delivery [7].

Micro- or nano-sized contrast agents entrapping gas within a surfactant-based shell oscillate through cycles of expansion and contraction, this in response to ultrasound. In this context ultrasound can also be used to rupture/implode the bubbles by a phenomenon known as inertial cavitation, which can generate microjets resulting in the propulsion of co-delivered therapeutics deep into surrounding cells/tissue [3]. This approach has met with some success in posterior eye drug delivery, with improved molecular penetration through successive layers of the retina demonstrated both *in vitro* and *ex vivo* [8–10]. That said translation of micro/nanobubbles and ultrasound as a modality for effective and reproducible drug delivery has been hampered due to the heterogeneity and instability of reported formulations [11, 12]. As the efficacy and reproducibility of ultrasound-triggered bubble rupture is highly dependent on these factors, we first addressed and optimised both vesicle size and formulation stability in order to improve the translational potential and reliability of the approach [13–18]. Furthermore, earlier *in vitro* studies investigating micro/nanobubbles have failed to grasp the complex nature of the multicellular retina, evaluating only the effects on a single cell type/population. Given the likely differences in intercellular sensitivity to the effects of ultrasound-assisted bubble cavitation, broader evaluation of co-localised cell types is expected to provide a more holistic understanding of the impact that ultrasound-assisted administration of our nanobubbles will have on representative cells of the retina [4, 5, 8–10, 19].

First, the development of a process to generate a highly stable, echogenic and homogeneous (*c*.*f*. commercial ‘microbubble’ formulations) nanobubble formulation was achieved [20]. Next, systematic evaluations centred on determining cell viability following incubation with nanobubbles and ultrasound application. In the absence of any observable cytotoxicity we performed a heat shock protein assay, to assess whether their levels were elevated. Finally, following optimisation of ultrasound-nanobubble treatment across all cell types, we demonstrated enhanced delivery of a model (whole) IgG antibody to three distinct yet clinically relevant retinal cell lines.

## Materials and methods

### Materials

1,2-dipalmitoyl-*sn*-glycero-3-phosphocholine (DPPC) and 1,2-distearoyl-*sn*-glycero-3-phosphoethanolamine-N-[methoxy(polyethylene glycol)-2000] (DSPE-PEG(2k)-OMe) were purchased from Avanti^®^ Polar Lipids (Alabaster, AL). Biotinylated IgG α-rabbit antibody (BA-1000) was purchased from Vector Laboratories, Ltd. Streptavidin-Cy3 was purchased from Life Technologies^™^. Perfluoropropane (PFP) was purchased from Coregas (Darra, QLD, Australia). Thiazolyl blue tetrazolium bromide (MTT), trypan blue and bovine serum albumin (BSA) were purchased from Sigma Aldrich^®^. Heat shock protein 70 (HSP70) antibody (#4782) was purchased from Cell Signaling Technology^®^.

All three cell lines were kindly donated by various institutions specialising in ocular research. ARPE-19 cells were obtained from A/Prof Damien Harkin (Queensland Eye Institute, Brisbane, Australia). The Moorfields-Institute of Ophthalmology human Müller cell line-1 (MIO-M1) [21] was used in the study. This was provided by GA Limb from the UCL Institute of Ophthalmology, London, UK. 661W cells were obtained from Dr Riccardo Natoli (John Curtin School of Medical Research, Australian National University, Canberra, Australia).

### Formulation of nanobubbles

Nanobubbles were prepared using a formulation strategy adapted from earlier published methods [20]. Briefly, liposomes prepared using DPPC and DSPE-PEG(2k)-OMe (94:6 molar ratio) were placed in an air evacuated crimp sealed vial. PFP was then added via gas tight syringe, pressurising the liposomes, which facilitated entrapment of contrast agent. Formulations were subsequently downsized into nanobubbles using established liposomal size standardisation techniques. The nanobubbles were compared to a reference conventional bubble formulation with an identical lipid and gas composition which was prepared using published methods [22].

### Evaluation of nanobubble size and homogeneity

Nanobubble size and polydispersity were evaluated for up to 56 days using dynamic light scattering (Zetasizer Nano ZS, Malvern, UK). Readings were taken at 4, 25 and 37°C to evaluate the impact of temperature on nanobubble stability.

### Evaluation of echogenicity

Grayscale contrast imaging was performed using a clinical Ellex^®^ Eyecubed ultrasound unit equipped with a 10 MHz probe (Ellex Medical, Adelaide, Australia). Formulations were injected into a polypropylene phantom filled with PBS prior to evaluation. Images were taken in B-Scan mode (1550 m/s, Log 65 dB + TGC).

### Microscopic evaluation of nanobubble morphology

The formulation was imaged using cryo-transmission electron microscopy (Cryo-TEM). An aliquot (4 μL) of the bubble formulation was transferred onto C-flat holey carbon grids in an FEI Vitrobot Mark III (FEI Company, Eindhoven, The Netherlands), with the chamber set to 4°C and 100% humidity. An optimal blot time of 3–4 seconds was employed, followed by plunging of the sample into liquid ethane. Frozen/vitrified samples were viewed on a Technai^™^ F30 TEM (FEI Company) operating at 300 kV, and imaged at 39,000x magnification (2x binned) with a Direct Electron^®^ LC1100 4k x 4k camera (Direct Electron^®^, San Diego, USA), using low-dose mode of SerialEM image acquisition software (http://bio3d.colorado.edu/SerialEM/). The defocus was set to -2.0 μm; with an electron dose of ~30 e-/Å^2^ (electrons/angstrom squared).

### Therapeutic ultrasound administration

An off-the-shelf portable ultrasound unit (JUS-2, Johari^®^ Digital, Jodhpur, India) equipped with a 38 mm diameter face plate (25 mm diameter transducing piezo crystal) and operated at 1 MHz with the option to employ pulse (10, 20, or 50%) or continuous wave administration, at intensities ranging from 0–2.5 W/cm^2^ (in 0.5 W/cm^2^ increments) was utilised. The probe face was always placed directly under the cell culture plate, with coupling gel liberally applied prior to ultrasound probe positioning and activation.

### Cell culture

ARPE-19 (retinal pigment epithelium, human origin) cells were cultured in DMEM/F12 medium. MIO-M1 (Müller glia, human origin) and 661W (photoreceptor, mouse origin) cells were cultured in DMEM. All media was supplemented with 10% FBS and 1% penicillin/streptomycin. Cells were incubated at 37°C in a 5% CO_2_/95% air environment.

### Assay of ultrasound and nanobubble toxicity

The MTT assay was utilised to evaluate the long term effects of nanobubbles and ultrasound on cell viability. Cells were cultured in 24-well plates and grown to 90% confluency prior to commencing studies. Ultrasound and nanobubbles were administered either alone or in combination to determine the safety of each technique.

Cells were incubated for 24 hours at 37°C in a 5% CO_2_/95% air environment subsequent to ultrasound and/or nanobubble administration. Following incubation, MTT was added to the media to a final concentration of 0.05% w/v and the cells were incubated for another 3 hours to allow for formazan production. Media was subsequently removed and the formazan crystals dissolved in DMSO. Aliquots (100 μL) of dissolved formazan were transferred to 96-well plates and fluorescence was evaluated at 595 nm using a plate reader (Bio-Rad, Hercules, CA, USA).

### Assay of heat-induced cellular stress

Levels of heat shock protein 70 (HSP70) generated as a result of optimised ultrasound application were evaluated via western blotting. Cells were grown to 90% confluence and subjected to 30 seconds of ultrasound (1 MHz, 20% duty, 0.5 W/cm^2^) in presence of the nanobubbles (30 μg/mL). Protein content was assayed 4, 8 and 24 hours following ultrasound application.

#### Protein extraction

Following incubation, the cells were bathed in sterile PBS. After the wash, PBS was aspirated before cells were lysed with 150 μL RIPA-containing lysis buffer with protease inhibitor (cOmplete^™^, Mini, EDTA-free, Roche) on ice for 20 minutes. The suspension was harvested and centrifuged at 12,000 *g* for 10 minutes after which the supernatant was aspirated with care ensuring the pellet containing unwanted debris remained undisturbed. Protein content was quantified using the Pierce^™^ BCA Protein Assay kit (Thermo Scientific, Inc., IL, USA). In preparation for western blot analysis, protein lysates in suspension were mixed thoroughly with sodium dodecyl sulphate (SDS) loading buffer at a 4:1 dilution of protein to loading buffer. Samples were boiled at 95°C for 5 minutes before vortexing and brief centrifugation to harvest residues.

#### SDS-PAGE gel electrophoresis

Sodium dodecyl sulphate polyacrylamide gel electrophoresis (SDS-PAGE) was employed to determine HSP70 expression levels. Briefly, samples and the protein ladder were loaded whilst gels were submerged fully in SDS-PAGE running buffer and run at 120 V for 30 minutes. Proteins were transferred to an Immobilon^™^-FL PVDF membrane (Millipore, UK) which was activated in methanol for 10 seconds before washing in transfer buffer. Membrane and gel complex were secured and run at 350 mA for 1 hour. Protein transfer was confirmed by means of protein ladder (Fermentas, UK) identification upon the membrane.

#### Western blot analysis

All steps in the following section were carried out at room temperature. Protein samples transferred to the PVDF membrane were blocked by use of 5% BSA in PBS-T blocking buffer for 1 hour. The membrane was washed with PBS-T three times for 10 minutes each time on a rocking bed. Antibody probing was carried out in 1% BSA-TBS-T blocking buffer at 1:1000 HSP70 and 1:2000 β-actin (Abcam^®^, ab13822, polyclonal, chicken-α-human) antibody dilutions. Three wash steps were performed again before administration of secondary conjugated LI-COR^®^ antibodies (Cambridge, UK) for 30 minutes away from light. Three final wash steps were carried out, away from light, before the membrane was scanned and analysed with an Odyssey^®^ Infrared Imaging System (LI-COR^®^, Nebraska, USA). The control ratio of HSP70 to β-actin was calculated and normalised.

### Antibody uptake into retinal cells assisted by nanobubbles and ultrasound

The vast majority of both current and investigative therapeutics developed to treat retinal pathology are biological macromolecules, which require prompt internalisation by cells/tissues in order to demonstrate efficacy [23, 24]. With rapid and safe delivery our primary goal, we sought to investigate whether the nanobubble-ultrasound strategy promotes internalisation of a model IgG-based macromolecule into clinically relevant cell types. To assess this, cells were cultured on glass coverslips in 24-well plates and allowed to grow to 90% confluency, and subsequently incubated with nanobubbles followed by optimised ultrasound application to promote intracellular delivery of a co-incubated biotinylated α-rabbit antibody (BA1000, IgG). The model therapeutic was either administered alone or mixed with nanobubbles just prior to addition to the wells at an antibody concentration of 50 μg/mL. Following incubation (2 minutes) in the presence of nanobubbles and subsequent administration of ultrasound (20 or 30 seconds), the cells were incubated for a further 2 hours at 37°C in a 5% CO_2_/95% air environment. Cells were then washed with PBS before fixing with ice cold methanol and permeabilisation using 0.1% v/v Triton-X100. Next, Cy3-streptavidin was added to detect for biotinylated antibody, with coverslips mounted on slides using DAPI-containing mounting medium. Antibody uptake was initially evaluated using fluorescence microscopy (NIKON^®^ Eclipse Ti-E) and subsequently confirmed using confocal microscopy (Olympus Fluoview^®^ FV1200). Quantitative analysis of uptake was achieved using fluorescence microscopy, where 5 images per slide were taken at set regions (centre, top, bottom, left and right fields), with mean Cy3 fluorescence intensity per cell nucleus evaluated using Image-J software (http://imagej.nih.gov/ij/).

### Statistical analysis

All values are shown as mean ± SD following experiments carried out at n≥3. Comparisons among groups were conducted using one-way or two-way ANOVA which featured a Dunnett’s multiple comparisons post-test, with p<0.05 being considered statistically significant.

## Results

### Nanobubbles offer a highly echogenic and shelf-stable alternative to conventional bubble formulations

To-date, a fundamental limitation of commercially available micro/nanobubble formulations has been their heterogeneous bubble size [11]. Our optimised nanobubble formulation strategy significantly reduced bubble size and heterogeneity, with highly echogenic nano-sized vesicles retained for up to eight weeks under optimised storage conditions (Table 1). Conversely, conventional bubbles were both significantly larger and highly heterogeneous throughout the course of the eight week stability study; this despite a predominantly nanosized population existing in both cases. Optimised nanobubbles also appeared to be more stable upon visual observation whereas the conventional bubble formulation showed sedimentation and caking over the storage period.

**Table 1:** 56 day Z-average size and polydispersity index profiles of standard and optimised nanobubbles.

	Conventional DPPC bubbles		Optimised DPPC nanobubbles	
	*Z*-ave (nm)	PDI	*Z*-ave (nm)	PDI
Day 1	606.4 ± 132.4	0.496 ± 0.096	205.0 ± 35.3	0.331 ± 0.095
Day 28	522.3 ± 48.2	0.470 ± 0.050	219.3 ± 43.4	0.352 ± 0.094
Day 56	439.8 ± 55.0	0.388 ± 0.069	211.2 ± 64.5	0.354 ± 0.035

### B-scan ultrasound confirms enhancement and retention of nanobubble echogenicity

**Fig 1:**
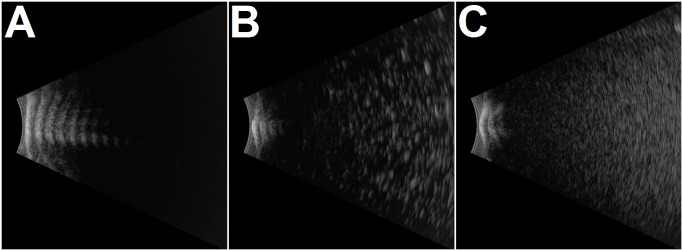
B-scan ultrasound images obtained after visualising (A) liposomes, (B) conventional nanobubbles and (C) optimised nanobubbles using diagnostic ultrasound.

Gaseous particles are readily visualised on B-scan ultrasound, appearing as white contrast, with the intensity of contrast correlating with the amount of gas present. From visual appearance alone the optimised nanobubbles demonstrated a superior echogenicity to conventional bubbles, suggesting that the novel preparation process improved gas entrapment. B-Scan images were in agreement with visual observations, with the optimised particle (Fig 1C) demonstrating superior contrast to standard (Fig 1B) nanobubbles. A complete absence of signal when plain liposomes were imaged confirmed the sensitivity of this analysis to the presence of gas in-formulation (Fig 1A).

### Nanobubbles resemble their liposomal precursors

**Fig 2:**
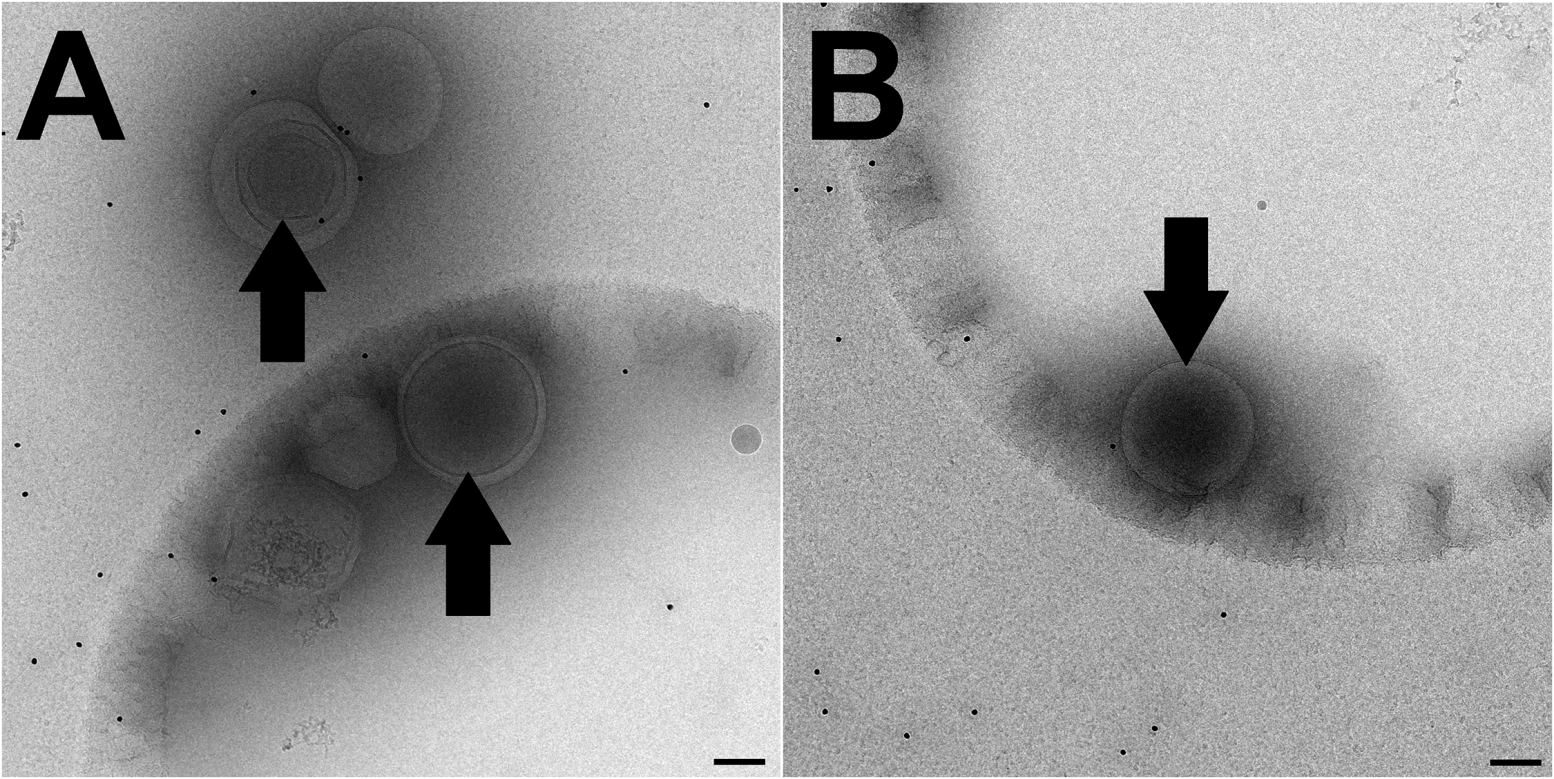
Cryo-TEM images of nanobubble formulation. (A) Population of five nanobubbles showing varied size and lamellarity, (B) Single nanobubble with strong dark colouration. Arrows point to bubbles with evidence of dark luminal colouration. Scale bar 100 nm.

Optimised nanobubbles were observed via TEM as spherical nanovesicles with dark lumina and absence of aggregation (Fig 2). Obtained parameters of particle size and homogeneity were in agreement with values obtained via other means of size characterisation. The prepared vesicles varied in lamellarity and luminal darkness (Fig 2A), both of which generally appeared to increase with increasing particle size.

### Ultrasound-nanobubble combination toxicity is consistent across retinal cell lines

**Fig 3:**
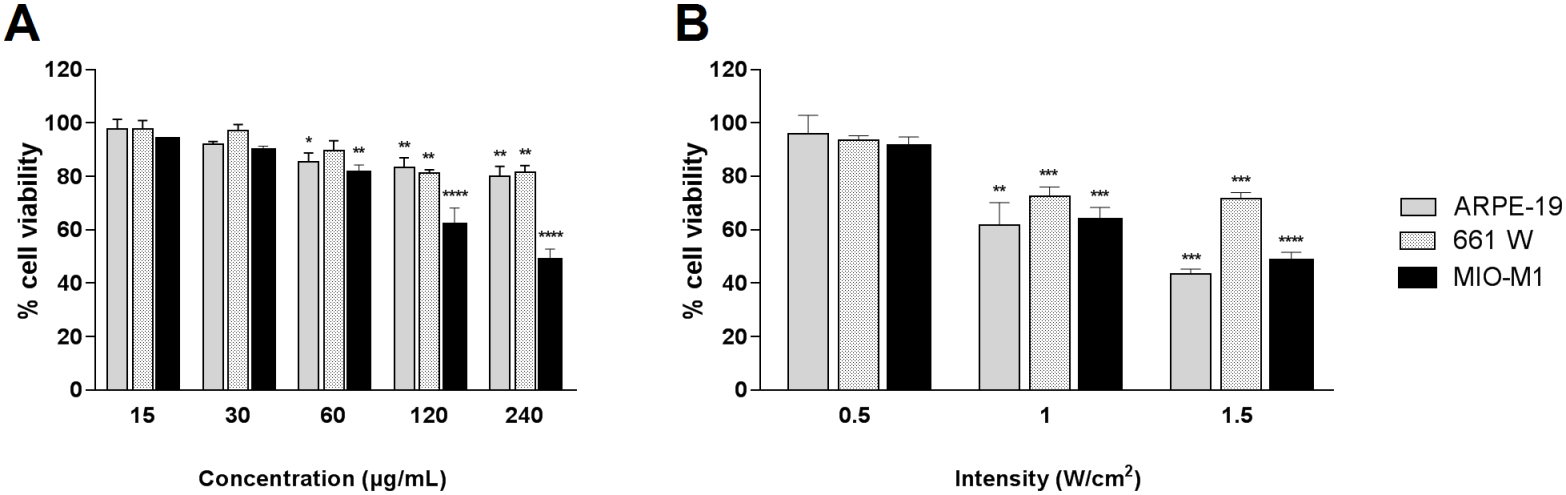
Comparative viability of three unique retinal cell lines as determined by the MTT assay. (A) Concentration-dependent toxicity of nanobubbles; (B) Combination nanobubble and ultrasound toxicity with nanobubble concentration fixed at 30 μg/mL. In all cases of ultrasound exposure; frequency, duty cycle and administration time were fixed at 1 MHz, 20% and 30 seconds, respectively. Conditions under which viability of a cell line is significantly lower than that in absence of any treatment have been identified (one-way ANOVA).

ARPE-19 and MIO-M1 cell lines were most susceptible to concentration dependent nanobubble toxicity, with a significant decrease in viability noted at concentrations of 60 μg/mL and above (p<0.05, Fig 3A). 661W cells were more resilient, only showing toxicity at nanobubble doses of 120 μg/mL and above. Interestingly, when ultrasound was combined with nanobubbles (at 30 μg/mL) all three cell lines showed signs of toxicity at identical levels of stimulus application. To elaborate, an ultrasound intensity of 1 W/cm^2^ (1 MHz, 20% duty cycle, 30 seconds) applied to cells in presence of 30 μg/mL nanobubbles significantly reduced viability across all cell types, however halving the intensity to 0.5 W/cm^2^ saw cell viability unaffected (Fig 3B).

### Heat stress studies reveal no HSP70 upregulation following administration of optimised ultrasound

**Fig 4:**
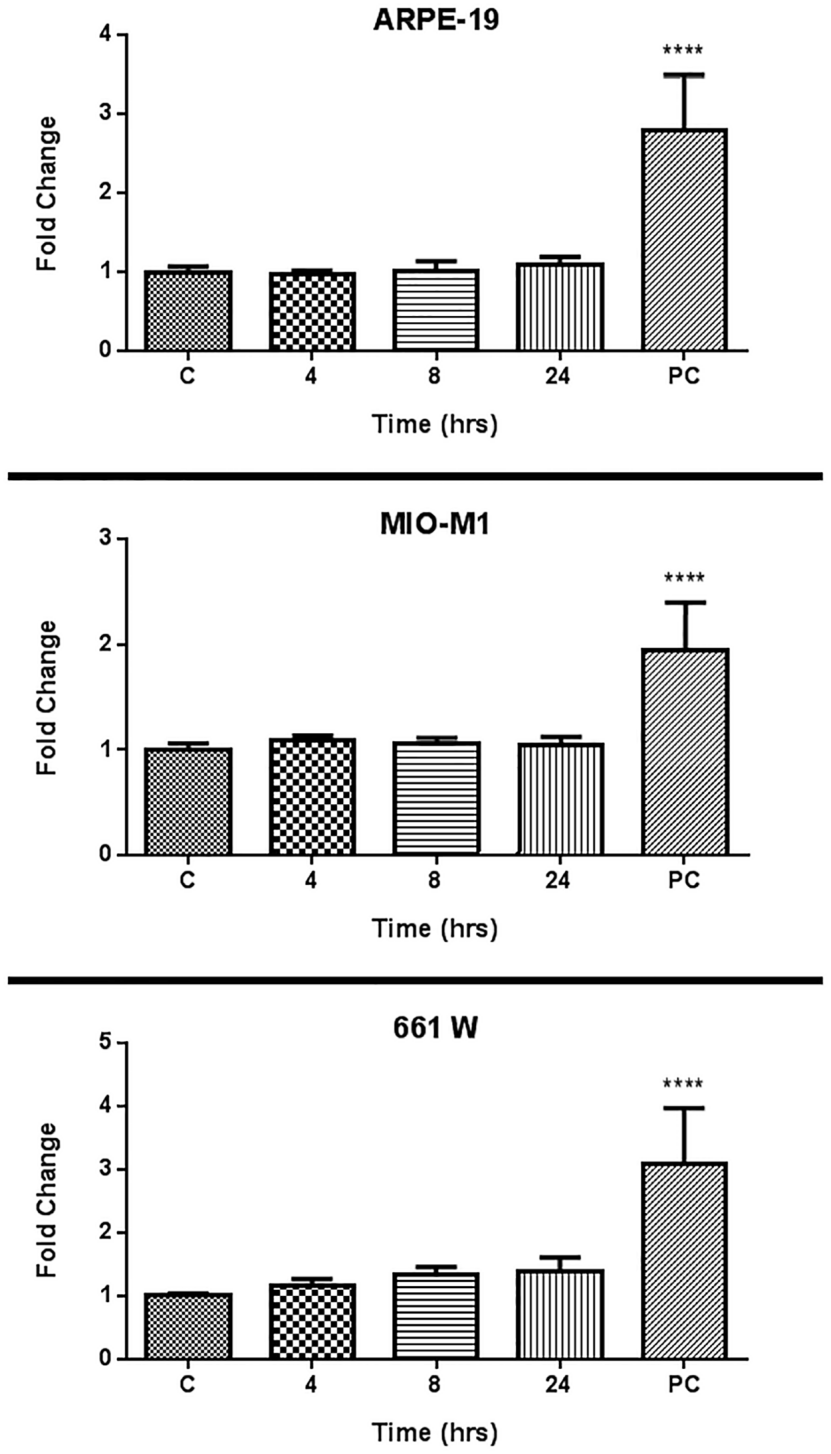
Normalised ratio of HSP70 to β-actin after set time points following administration of ultrasound (1 MHz, 20% duty cycle, 0.5 W/cm^2^, 30 seconds) in presence of 30 μg/mL nanobubbles to (Top) ARPE-19, (Middle) MIO-M1, and (Bottom) 661W cells (n = 3). C = control, PC = positive control (1 MHz, 20% duty cycle, 1.0 W/cm^2^, 30 seconds). Samples were analysed using one-way ANOVA.

An ultrasound-nanobubble combination deemed toxic on cell lines in earlier viability studies was utilised as the positive control in this analysis. Here, HSP70 levels were elevated for all three cell types when compared to the control (Fig 4, p<0.0001). When HSP70 levels were assessed using a safety-optimised ultrasound protocol, no significant upregulation was observable on any of the cell lines at any of the tested time points (Fig 4).

### A clinically relevant macromolecule can be effectively delivered to various retinal cells using a nanobubble-ultrasound combination

**Fig 5:**
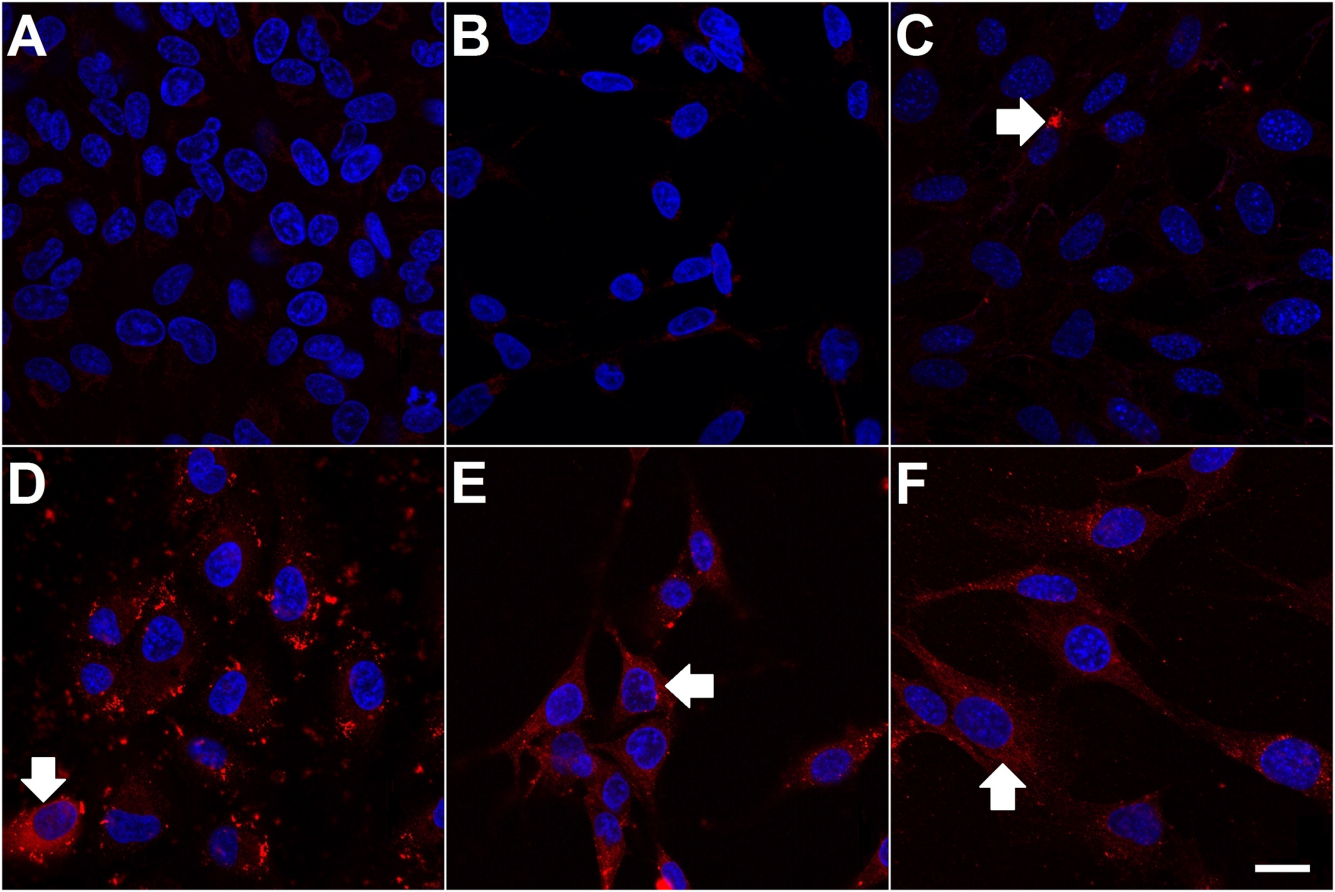
Sample confocal microscopy images of ARPE-19 cells administered (A) antibody only and (D) antibody in the presence of ultrasound and nanobubbles; MIO-M1 cells administered (B) antibody only and (E) antibody in the presence of ultrasound and nanobubbles; 661W cells administered (C) antibody only and (F) antibody in the presence of ultrasound and nanobubbles. Arrows indicate a cell in which antibody uptake (red) was observed. Scale bar = 20 μm.

Biotinylated IgG antibody internalisation was observed in all three cell populations via confocal microscopy (Fig 5). Cy3-streptavidin detection of the internalised biotinylated antibody was generally very sparing in instances where only antibody was incubated with cells in absence of any other treatment (Fig 5A, 5B and 5C). In contrast, groups administered an ultrasound-nanobubble combination showed a high proportion of cells with cytoplasmic Cy3-streptavidin (Fig 5D, 5E and 5F).

**Fig 6:**
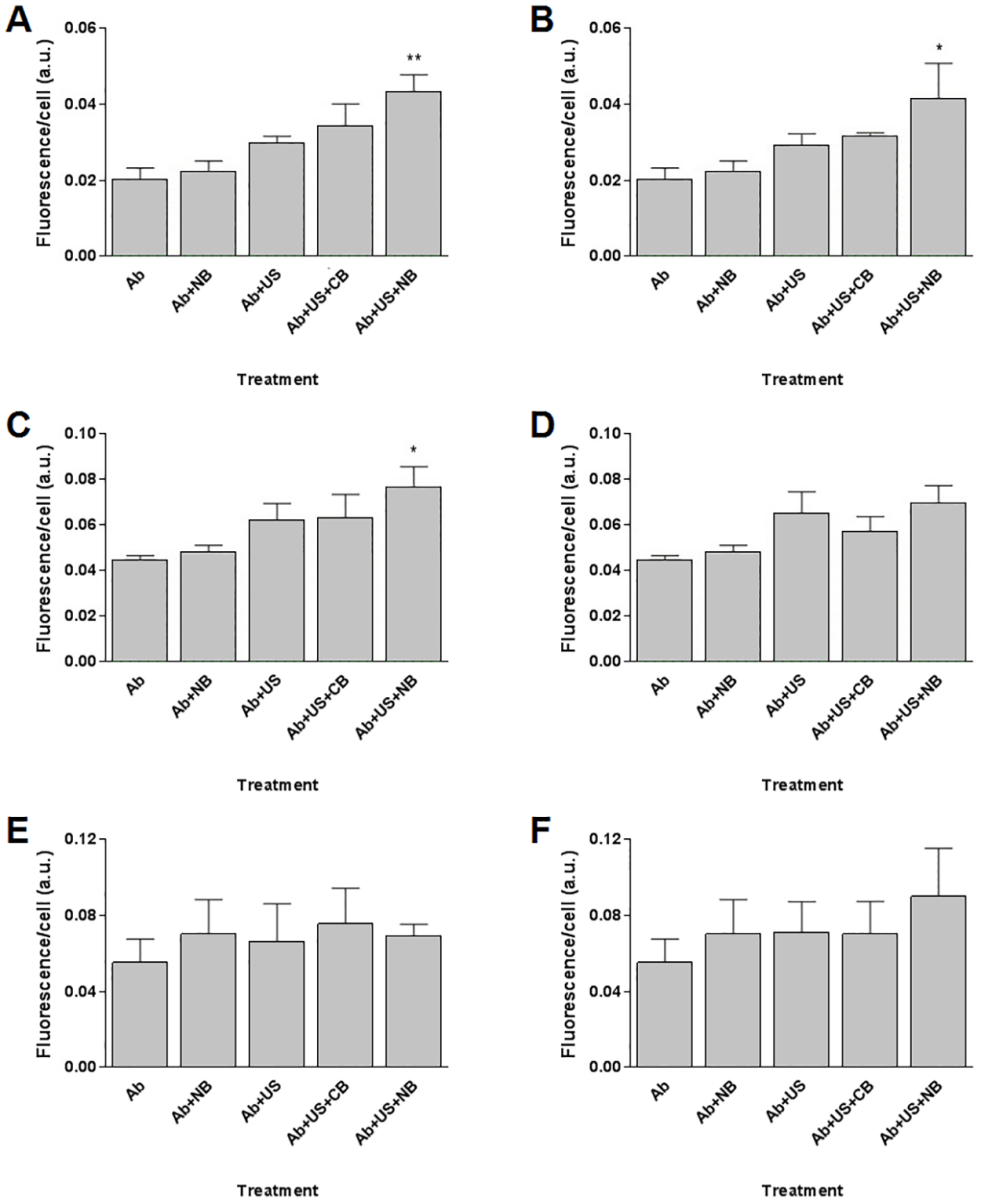
Fluorescence per cell observed following various biotinylated antibody administration protocols. Top: ARPE-19 cell lines administered (A) 1.0 W/cm^2^ for 20 seconds, (B) 0.5 W/cm^2^ for 30 seconds. Middle: MIO-M1 cells administered (C) 1.0 W/cm^2^ for 20 seconds, (D) 0.5 W/cm^2^ for 30 seconds. Bottom: 661W cells administered (E) 1.0 W/cm^2^ for 20 seconds, (F) 0.5 W/cm^2^ for 30 seconds. In all cases, the ultrasound frequency and duty cycle were fixed at 1 MHz and 20%, respectively. Key: Ab = antibody, NB = optimised nanobubble formulation, US = ultrasound, CB = minimally echogenic control bubble formulation, a.u. = arbitrary units. Samples were analysed using one-way ANOVA.

With safety of two unique ultrasound protocols confirmed, both were applied for 20 or 30 second duration in the presence of optimised nanobubbles (30 μg/mL) to evaluate antibody uptake and quantification in all three cell lines. A formulation of lipid bubbles virtually devoid of gas and representative of conventional bubble (CB) formulations was utilised as a control. In all cases, fluorescence per cell only increased significantly in instances where ultrasound was used in combination with optimised nanobubbles (Fig 6). Co-delivery of antibody and nanobubbles in the absence of ultrasound had no impact on macromolecular uptake, nor did application of ultrasound in the absence of bubble formulations. Combining antibody with CB and ultrasound also failed to increase mean fluorescence per cell (p>0.05).

Neither of the tested nanobubble-ultrasound protocols was able to demonstrate antibody uptake in 661W cells (Fig 6E and 6F). In the case of MIO-M1 cells, applying the higher intensity of 1.0 W/cm^2^ for 20 seconds in presence of nanobubbles significantly increased antibody uptake (Fig 6C, p<0.05) whereas applying the lower intensity of 0.5 W/cm^2^ for 30 seconds to the combination caused no observable difference in uptake (Fig 6D). Finally, for ARPE-19 cells, both tested conditions of ultrasound administration in combination with nanobubbles significantly increased macromolecule uptake (p<0.01 using 1.0 W/cm^2^ for 20 seconds [Fig 6A], p<0.05 using 0.5 W/cm^2^ for 30 seconds [Fig 6B]).

## Discussion

The focus of this study was to evaluate the toxicity and validate the utility of ultrasound-triggered nanobubble delivery of a macromolecule in representative cells of the retina. As ultrasound administration will likely subject a broad region of the tissue to similar mechanical and thermal stressors, we conducted evaluations on three different cell lines which represented unique populations found within the retina i.e. epithelial (ARPE-19), glial (MIO-M1), and neuronal (661W) cell types.

To address shortcomings with existing, commercially available bubble formulations we first elected to develop a highly stable and echogenic nanobubble formulation for subsequent evaluation *in vitro*. Although commonly overlooked in the case of microbubble delivery systems, size standardisation is crucial to ensure ultrasound-triggered drug delivery is reproducible. Ultrasound responsiveness is closely related to particle size and composition; hence a more homogenous formulation is expected to yield highly reproducible and predictable drug delivery [13–16]. Through systematic development and optimisation of our ‘in-house’ formulation process, we generated a homogenous and intensely echogenic nanosized bubble formulation. While this follows an increasing trend of nanosized bubble formulations being reported in literature [16, 25–31], a key concern regarding bubbles of this nature has been their echogenic stability, it being anticipated that gas will not be effectively retained within nanosized particles [32]. In contrast our formulation possesses highly desirable storage and *in vitro* stability from the context of echogenicity, and we propose this to be the result of more refined bubble processing as well as storage conditions being tailored to the formulation.

Coming to the focus of this study, next we sought to determine both the safety and efficacy of nanobubbles and ultrasound in three *in vitro* models of the retina. Previous studies have elucidated that ultrasound-induced permeabilisation may be detrimental to cell health. For instance, electron microscopy has shown that ultrasonic cavitation causes superficial wounds on the surface of cells [33]. Separately, ultrasound-induced heat stress has also been identified as a trigger for intracellular inflammation [34]. Given the serious implications of these effects, multiple assays were utilised to determine safety of the technique. The MTT assay is among the most commonly employed indicators of cellular viability. Although previously questioned for its validity when used in conjunction with lipid based formulations [35], the assay provided robust and reproducible data for our viability evaluations and was pursued as the assay of choice. Nanobubbles and ultrasound affected viability in a predictable concentration- and exposure-dependent manner, respectively. Interestingly, when cell death was assessed using trypan blue uptake immediately following ultrasound exposure (data not shown), these values were in very close agreement to the MTT data obtained 24 hours later. This suggests that ultrasound-mediated toxicity predominantly occurred in the acute phase with negligible longer term detrimental effects to cells.

When comparing the degree of toxicity induced upon three cell lines by each of the nanobubble formulations alone and the nanobubble-ultrasound combination, it became apparent that each cell type responded differently, most notably in the case of 661W cells, which proved to be the most resistant to nanobubble induced toxicity. However, analysis of toxicity imparted by nanobubble-ultrasound in combination showed that all cells demonstrated identical toxicity thresholds. Being a non-discriminatory technique that facilitates drug uptake by means of cell bilayer disruption rather than specific receptor interactions, it was encouraging to note that toxicity is predictable irrespective of retinal cell type.

It is well accepted that ultrasonic energy above a threshold readily generates heat, which may be detrimental to cell health [36]. The cellular response to an ultrasound-driven increase in localised temperature has not been extensively evaluated for long-term effects [34]. Heat shock proteins are a family of highly conserved mediators which serve various chaperoning functions in response to heat stress [37]. An absence of HSP70 up-regulation during optimised nanobubble-ultrasound conditions could be clearly contrasted with the positive control, further confirming the safety attained following optimisation of our administration strategy. Interestingly, the apparent overlapping trends observed with trypan blue, MTT and HSP70 evaluations on cell health suggest that a single viability evaluation may be adequate in the future to determine the toxicity of nanobubble-ultrasound administration on a given cell population.

Ultrasound-associated cellular uptake of macromolecules has previously been reported with pDNA [9, 38] and also fluorescently-labelled carriers [4] serving as markers. Our ultimate aim here was to efficiently and rapidly facilitate macromolecule internalisation, and to assess this we employed a biotinylated antibody that could be readily detected with Cy3-labelled streptavidin after fixation, for quantification purposes. Due to natural flow mechanisms that exist within the vitreous, an injected therapeutic may only be retained proximal to the retina transiently [39]. In absence of ultrasound and nanobubbles, the macromolecule had a very low tendency to associate with or be internalised by the three cells of interest over the two hour incubation period. The degree of internalisation varied by cell type and for two cell lines (i.e. ARPE-19 and MIO-M1), utilising the nanobubble-ultrasound combination enhanced antibody delivery, as visualised with both fluorescence and confocal microscopy. Ultrasonic cavitation is reported to create large (up to several micron) [40] yet transient (1–2 minute lifespan) pores on the membrane of cells [41]. While an antibody-sized macromolecule can traverse through a channel of such dimensions, effective permeation will rely on an adequate number of pores forming on the cell surface coupled with a driving force promoting cellular internalisation. Ultrasound alone had no significant impact on antibody uptake into each cell type, whereas combining this with our contrast agent-rich nanobubbles was the key driver of antibody internalisation. These findings are in line with the well-known mechanism of micro/nanobubble-assisted molecular internalisation following ultrasonic exposure [7]. What remains unclear is why the technique failed to increase uptake in 661W cells, with this population otherwise responding to the ultrasound-nanobubble combination in a very similar fashion to the other two tested cell types. Current knowledge of the physiology and functionality of these cells alone cannot explain the observed differences and further evaluations are required to elucidate the role cellular structure, morphology and origin play in susceptibility to cavitation-induced delivery.

The selected cell types are very distinct in terms of their morphology and functionality both *in vitro* and within the intact neurosensory retina [42]. Each cell type is also affected in a unique manner in various posterior eye complications, namely neovascular diseases such as AMD and DR. Given its proximity to the highly vascular choroid layer and important age-related changes in cellular morphology, dysfunction of the retinal pigment epithelium (ARPE-19) is implicated in early stages of both diseases [43–45]. Müller cells (MIO-M1) also have a pivotal role in neovascular retinal diseases: their overproduction of VEGF being a key mediator that facilitates disease progression [46, 47]. Photoreceptor cells (661W) are also affected in both disease states, however as damage to the pigment epithelium precedes photoreceptor degradation [48, 49] these cells have received little attention as a primary target for early disease intervention. Taking these observations into consideration, it is possible that our optimised nanobubbles and ultrasound may be able to enhance therapeutic delivery to relevant cells implicated in earlier stages of neovascular diseases (ARPE-19 and MIO-M1) without having any impact on other localised cells subjected to the same treatment conditions (661W). However, due to differences in cellular origins (ARPE-19 and MIO-M1 being human derived whereas 661W are derived from mice), this observation may not stand when treatments are applied into a wholly human model.

All studies described herein were performed using an off-the-shelf, generic ultrasonic transducer probe. It is expected that, following the development of a purpose built transducer, effectiveness of this strategy can be further optimised, and drug delivery markedly improved [50–53]. Further refinements may include associating the nanobubbles with the therapeutic either by means of entrapment, conjugation or charge-based association; this proximity between contrast agent and therapeutic offers steric protection and may consequently enhance efficacy [54, 55].

Cavitation behaviour is also closely related to micro/nanobubble size and an ‘ideal’ protocol will facilitate rapid on-demand destruction of the bubbles leading to deposition of therapeutic into target cells/tissue. In our study, nanobubbles were subjected to a 1 MHz ultrasonic frequency, which is in contrast to literature reports citing 3 MHz as a more suitable frequency for the disruption of nano-sized contrast agents [15, 16]. In line with current understanding in the field, only our highly echogenic nanobubbles were able to significantly enhance antibody uptake beyond baseline (p<0.05), while the conventional bubbles had no notable impact on uptake (p>0.05). Conventional liposomes and nanoparticles (devoid of gas) have previously demonstrated ultrasound responsive behaviour such as triggered release of drug contained within them [56], and even enhanced tissue uptake of the entire vesicle [5] following ultrasound administration. In our hands such outcomes of enhanced antibody uptake were not observed when trialled in combination with a conventional bubble formulation, likely due to the fact that we adopted an *in situ* co-formulation approach, with the drug not being entrapped/loaded or conjugated with the delivery system. Therefore any impact that ultrasound may have had on non-gaseous particles did not substantially affect the surrounding milieu.

Optimal ultrasonic cavitation is a function of several parameters, with some previous reports suggesting peak-to-peak negative pressure as the driving force for optimal cavitation behaviour [7, 57], however this parameter was not tuneable or attainable with our generic ultrasound unit. From the two optimised ultrasonic protocols trialled, it was apparent that using a higher intensity for a shorter duration had a more desirable impact on cellular uptake, with 1 W/cm^2^ for 20 seconds significantly enhancing delivery to both ARPE-19 and MIO-M1 cells, whereas 0.5 W/cm^2^ for 30 seconds enhanced delivery solely to the ARPE-19 population. There is scope to evaluate more closely the mechanical impact of ultrasonic energy on our nanobubbles in order to optimise safety and delivery efficiency, where each parameter may be more thoroughly monitored.

To our knowledge, our study is the first in which ultrasound-mediated drug delivery has been assessed on Müller and photoreceptor cell derivatives. It is also one of very few studies that utilise multiple retinal cell lines to evaluate the consequences of posterior eye drug delivery, one notable example being a study evaluating the cellular impact of bevacizumab [19]. With the observations made in our study, it is clear that a multicellular evaluation will enable the derivation of more robust data regarding the impact of any drug delivery strategy on the complex retina. Moving forward from these evaluations it is now imperative to assess the efficacy of this technology in *ex vivo* and *in vivo* models.

## Conclusion

This study evaluated the impact of a novel nanobubble-ultrasound strategy on three distinct retinal cell lines. Nanobubble size standardisation was achieved while retaining strong echogenic properties indicative of ultrasound responsiveness. The synergistic effect of nanobubbles and ultrasound impacted each cell type in a unique manner highlighting the importance of carrying out holistic evaluations to determine the safety and efficacy of the technique when used on highly complex tissues, such as the retina. In-formulation gas retention was the clear driver for enhancing ultrasound-assisted delivery of a co-delivered macromolecule into the cells; this only being achieved with our highly echogenic nanobubble formulation. Future studies will aim to translate these findings into *ex vivo* and *in vivo* models of the eye.

## Supporting information

S1 File Supplementary protocols. (DOCX)
