# Supplementary material for: Stably engineered nanobubbles and ultrasound - An effective platform for enhanced macromolecular delivery to representative cells of the retina
Source: PLoS One. 2017 May 25;12(5):e0178305. doi: 10.1371/journal.pone.0178305 (PMC5444814; doi:10.1371/journal.pone.0178305)
Supplement: S1 File — (DOCX) [file pone.0178305.s001.docx]

# **Supplementary protocols**

##### Buffers utilised in HSP70 quantification assay

The following buffer compositions were utilised in each assay.

**SDS Sample Buffer (5X)**

| Tris-HCl | 3.875 g or Tris-HCl (pH 6.8) |
| --- | --- |
| SDS | 10 g |
| Bromophenol Blue | 0.05 g |
| Glycerol | 50 mL |
| DTT 0.1M* | 12.5 mL |
| ddH_2_O>Total | 35 mL |
| Total | 100 mL |

*DTT added just prior to use on each aliquot of sample loading buffer to prevent freeze-thaw cycle-induced degradation. The sample buffer stock with DTT is acceptable for use on a number of freeze thaw cycles (final DTT concentration in sample buffer is 12.5 mM).

**RIPA (Radio Immuno Precipitation Assay) Cell Lysis Buffer (2X)**

|  | Final concentration | Volume |
| --- | --- | --- |
| Tris-HCl | 50 mM (pH 7.4) | 10 mL (250 mM) |
| NaCl | 100 mM | 10 mL (500 mM) |
| Sodium deoxycholate | 1% (500 mg) | 10ml (5%) |
| SDS | 1% | 10ml (5%) |
| Triton X-100 | 1% | 500 µL |
| Total (ddH_2_0) | 50 mL | 9 mL |

Prior to usage buffer was diluted to 1X using ddH_2_O with protease inhibitors and/or phosphatase inhibitors. Varying volumes were used for cell lysis, which were dependent upon factors including cell confluence and culture growth area.

*N.B. Tris-HCl is made up and used with dilution factor for total volume of RIPA buffer.*

**Transfer buffer:** Tris (basic) 25 mM, Glycine 192 mM and 20% (v/v) methanol

**SDS running buffer X5:** Tris (basic) 125 mM, 1.25 M Glycine and 0.5% (w/v) SDS

##### **Method of quantifying cell fluorescence**

1. Count the number of cells present in-figure
2. Open TRITC filter image in Image-J software
3. Convert image “Type” to 8-bit
4. Use “Background Correction” Plug-In to eliminate artefacts and standardise image (available at <http://rsb.info.nih.gov/ij/plugins/background.html>)
5. Omit any clear artefacts if still visible using “Freehand selections” tool
6. “Analyze” > “Measure”
7.
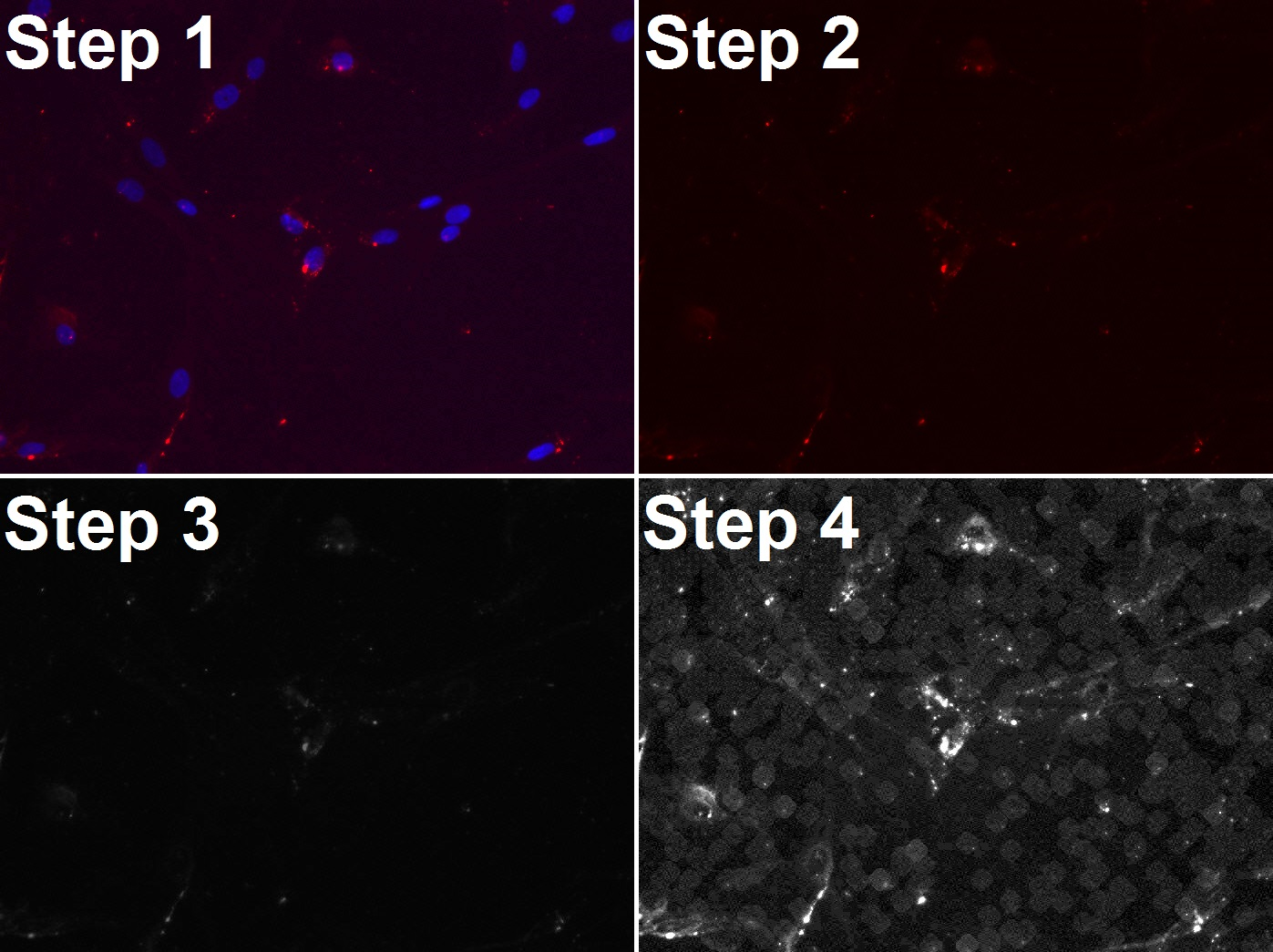
Divide obtained value by the number of cells to yield fluorescence/cell

**Fig S1. Image processing protocol for attainment of background corrected image.** Step numbers correspond to those mentioned above.

In the example presented in Fig S1, the process yielded a fluorescence value of 1.993 which was divided by the counted 23 cell nuclei (all partially visible nuclei were included in the analyses for consistency) for a “fluorescence/cell” value of 0.086652174. Background correction substantially improved quantification under grayscale settings.
